# Optimal Solubility of Diclofenac *β*-Cyclodextrin in Combination with Local Anaesthetics for Mesotherapy Applications

**DOI:** 10.1155/2017/8321325

**Published:** 2017-04-10

**Authors:** Giuseppe Tringali, Pierluigi Navarra

**Affiliations:** Institute of Pharmacology, Catholic University Medical School, Rome, Italy

## Abstract

Because of low injection volume, the recently marketed injectable solution of diclofenac in complex with *β*-cyclodextrin (Akis®, IBSA Farmaceutici Italia) is an ideal candidate for mesotherapy applications. In this study, we investigated the solubility of Akis, 25 and 50 mg/kg, in combination with various local anaesthetics (lidocaine, mepivacaine, bupivacaine, levobupivacaine, and ropivacaine) at different concentrations in aqueous vehicles (normal saline, sterile water, or bicarbonate). Final injection mixtures were classified as limpid, turbid, or milky at visual analysis under standardized conditions. We found that (i) the use of sterile water for injections or normal saline as vehicles to dilute Akis in combination with whatever local anaesthetic normally results in milky solutions and therefore is not recommended; (ii) using bicarbonate, optimal solubility was obtained combining Akis with lidocaine, both 1 and 2%, or mepivacaine, both 1 and 2%, whereas solutions were turbid in combination with bupivacaine, levobupivacaine, or ropivacaine. Thus, we recommend that Akis is used in combination with lidocaine or mepivacaine in a bicarbonate vehicle.

## 1. Introduction

By definition, mesotherapy refers to “the use of intra- or subcutaneous injections containing liquid mixture of compounds (pharmaceutical and homeopathic medications, plant extracts, vitamins, and other ingredients) to treat local medical and cosmetic conditions” [1]. Apart from cosmetic conditions and other procedures that might shed a controversial light on mesotherapy [1], the applications broadly including musculoskeletal pain (such as acute cervicalgia, lumbar pain, acute myositis, tendinitis, and traumatic disorders) are highly relevant in medical practice [2]. While the original mesotherapy approach involved the mandatory use of procaine, along with multiple (5 to 18) simultaneous injections with needles longer that 4 mm, the current approach is more reminiscent of a plain drug administration by local injection, with the notable difference of recommending low dosages [3]. As such, the advantages and drawbacks of mesotherapy are possibly the same of any local injective therapy, including enhanced local efficacy with reduced systemic exposure to drugs on the one hand and the issues of local tolerability (pain at the site of injection, skin reactions, etc.) on the other hand. On this regard, most important are the aspects of drug solubility and injection volumes, especially when mixtures of different active principles are to be injected.

The Company IBSA has recently marketed injectable solutions of diclofenac in complex with *β*-cyclodextrin; the formulation specifically developed for subcutaneous and intramuscular use was registered with brand name Akis. The higher solubility afforded by *β*-cyclodextrin allows us to limit the volume of injection to 1 mL, and 3 different strengths (25, 50, and 75 mg/vial) are available. Akis was shown to be bioequivalent to the comparator Voltaren® (a standard injectable solution of diclofenac sodium, 75 mg/3 mL) [4]. Akis of 25, 50, and 75 mg was found to be superior to placebo in a randomized double-blind trial for the treatment of pain associated with third molar extraction; both 50 and 75 mg were superior compared to placebo after 5 hours of drug administration, whereas 50 mg only remained superior after 6 hours [5]. The efficacy and safety of diclofenac *β*-cyclodextrin were also compared to those of diclofenac sodium or ketorolac in different paradigms of painful conditions, showing overall noninferiority or even superiority with respect to the comparator [for a review see [6]]. Because of the overall efficacy profile as an analgesic agent, as well as the reduced volume of injection and the availability of different strengths, Akis can be envisioned as a good candidate for mesotherapy applications.

In the present study, we have investigated the solubility of Akis in different vehicles, in combination with a variety of local anaesthetics; a practical approach was adopted to describe solubility, to provide useful suggestions for optimal preparation of injection solutions in the setting of mesotherapy applications.

## 2. Materials and Methods

The following drugs were tested: (1)* diclofenac β-cyclodextrin*: Akis 25 and 50 mg/mL strengths (Akis 75 mg/mL was not tested in the study because it proved to be not superior to the 50 mg formulation in efficacy trials; therefore, the higher amount of active principle in the 75 mg formulation would have only reduced the chance to obtain optimal dissolution, with no advantage in efficacy); (2)* lidocaine*: lidocaine hydrochloride: 10 mg/mL (1% W/V) [Lidocaina Cloridrato S.A.L.F.® 10 mg/mL]; lidocaine hydrochloride: 20 mg/mL (2% W/V) [Lidocaina Cloridrato Bioindustria® 20 mg/mL]; (3)* mepivacaine*: mepivacaine hydrochloride: 2% [Mepicain 2%® (Monico SPA)]; (4)* bupivacaine*: bupivacaine hydrochloride: 5 mg/mL (0.5% W/V) [Marcaina Iperbarica® 5 mg/mL (Astra Zeneca)]; bupivacaine hydrochloride: 5 mg/mL (0.5% W/V) [Marcaina® 5 mg/mL (Astra Zeneca)]; (5)* levobupivacaine*: levobupivacaine hydrochloride: 2,5 mg/mL (0,25% W/V) [Chirocaine® 2,5 mg/mL (AbbVie Srl)]; levobupivacaine hydrochloride: 7,5 mg/mL (0.75% W/V) [Chirocaine 7,5 mg/mL (AbbVie Srl)]; (6)* ropivacaine*: ropivacaine hydrochloride: 2 mg/mL (0.2% W/V) [Ropivacaina Kabi® 2 mg/mL (Fresenius Kabi)]; ropivacaine hydrochloride: 10 mg/mL (1% W/V) [Ropivacaina Molteni® 10 mg/mL (Molteni Farmaceutici)]. The above drugs were diluted to final volume injections in the following vehicles: sterile water for injection [Eurospital Spa]; normal saline [Eurospital Spa]; and sodium bicarbonate [Sodio Bicarbonato S.A.L.F. 10 mEq/10 mL].

To mimic what is feasible in daily clinical settings, the dilution procedure was made simple and results were based on a visual inspection only. Firstly 1 mL Akis solutions were diluted into the vehicle, and subsequently local anaesthetic solutions were added to a final volume of 5 mL, summarised in Table 1; the bicarbonate/local anaesthetic V/V ratio was always maintained within limits allowing optimal solubility, since bicarbonate in excess per se can induce precipitation [7].

**Figure 1:**
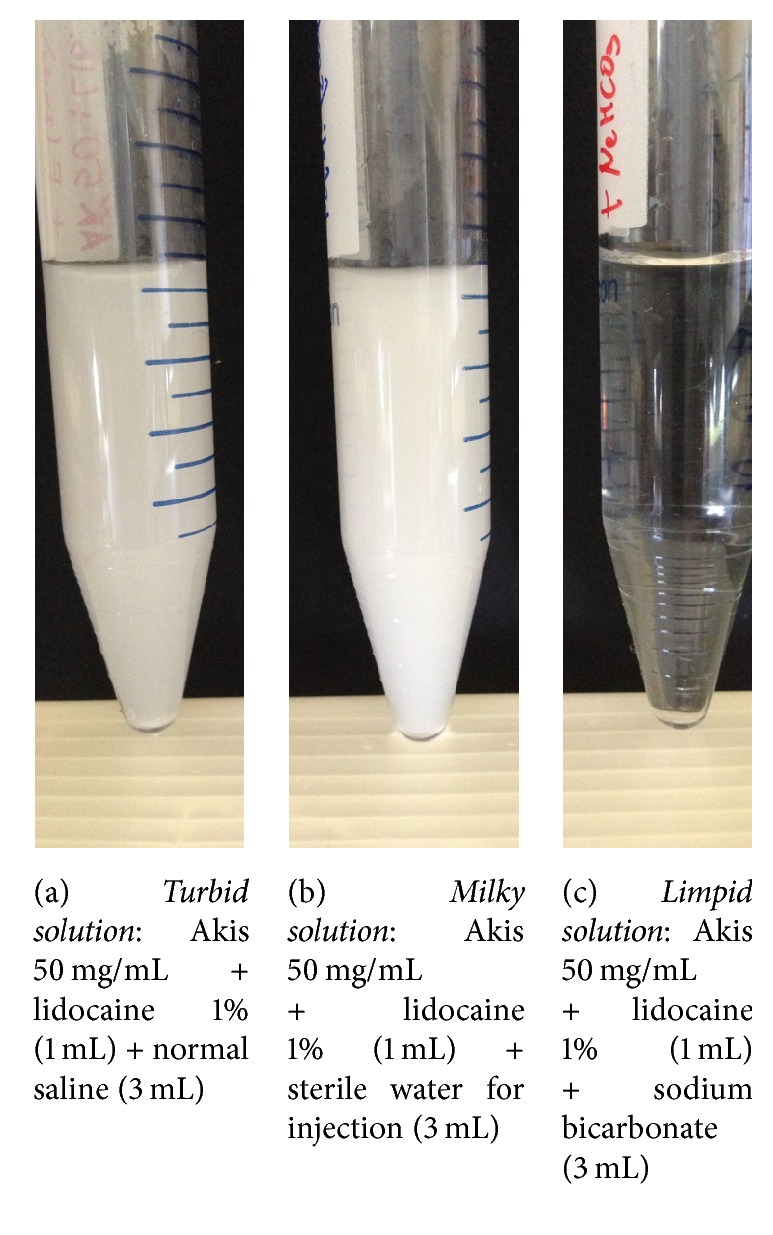
Representative images of a turbid (a), a milky (b), and a limpid solution (c). Volume of each mixture is reported in brackets.

**Figure 2:**
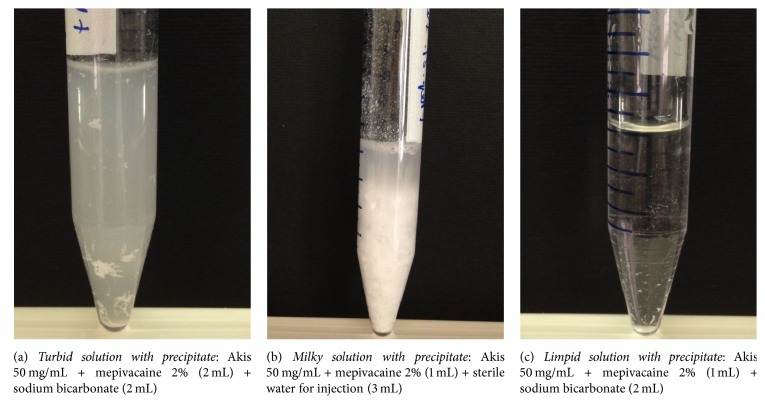
Representative images of a turbid (a), a milky (b), and a limpid solution with precipitate (c). Components of each mixture are reported in brackets.

**Figure 3:**
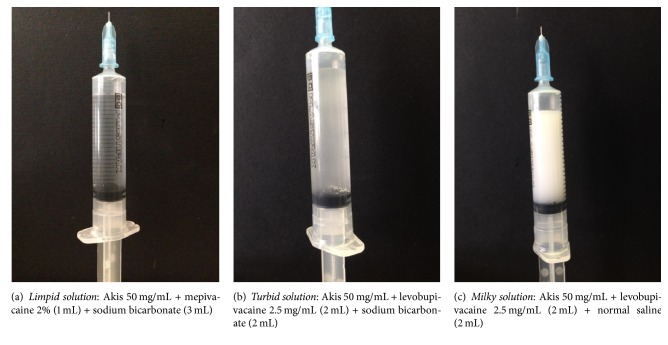
Representative images of a turbid (a), a milky (b), and a limpid solution (c) as they show in the syringe. Components of each mixture are reported in brackets.

The results of the visual inspection were classified into 3 broad categories: limpid solutions, turbid solutions, and milky solutions/suspensions. The occurrence of precipitates was also recorded. A number of representative examples of each category are shown in Figures 1–3, along with the cross reference to Table 1. Inspection was done immediately after preparation and after 24 hours. Given the descriptive nature of this study, no formal inferential statistics were carried out.

## 3. Results

All results are referred to freshly prepared solutions; after 24 hours, even freshly prepared limpid solutions tend to precipitate in most cases. Thus, an initial recommendation rising from this observation is to use freshly prepared solutions only.

Table 2 shows the combinations of vehicle, Akis, and anaesthetic that present limpid or turbid after preparation. Looking at these data, the following overall conclusions can be drawn:

1. Mixtures of Akis, both 25 and 50 mg/vial, with all anaesthetics tested result in milky solutions when diluted with sterile water for injection or normal saline; such preparations show milky solution, with or without a precipitate and/or solid aggregates on the vial walls. Therefore, the use of sterile water for injections or normal saline as vehicles to dilute Akis in combination with whatsoever local anaesthetic is not recommended.
2. At variance with sterile water and normal saline, using bicarbonate we obtained limpid solutions diluting Akis (both 25 and 50 mg strengths) with lidocaine, both 1 and 2%, or mepivacaine, both 1 and 2%, whereas solutions were turbid if the local anaesthetic was bupivacaine, levobupivacaine, or ropivacaine. Thus, lidocaine and mepivacaine are strongly recommended for association with Akis in a bicarbonate vehicle, at the final dilutions shown in Table 2; all other combinations leading to turbid solutions are not recommended.

## 4. Discussion of the Results

Here we showed that the combination of Akis with lidocaine or mepivacaine in a bicarbonate vehicle provides optimal results concerning drug solubility and the preparation of limpid solutions for injection, based on visual inspection. Concerning the choice of visual inspection as the end-point of measure, a thorough methodological approach would have suggested the use of a spectrophotometer to provide a quantitative measure of solution absorbance. From this point of view, the current end-point should be considered as a limitation of the study. However, since we had in mind to provide a practical guidance to the operators, the visual analysis has the obvious advantage to reproduce the real-life clinical setting.

We do not recommend sterile water or normal saline as vehicles for Akis + local anaesthetics mixtures, since such combinations normally lead to turbid and milky solutions, which may also be associated with the occurrence of bulky precipitates. Although injectable formulations presenting as suspensions sometimes are used in clinical practice, such formulations are specifically prepared to obtain a delayed absorption of active principles from the injection site, thereby prolonging the duration of systemic drug effects, whereas in the majority of cases in clinical practice it is well established that solutions for injection should present limpid in the syringe [8], in order to minimize the risk of local reactions to injection.

Lidocaine and mepivacaine share the same amidic chemical structure and display the same pKa value of 7.8. According to the Henderson-Hasselbalch equation, alkalinisation of the solution with bicarbonate causes an increase in the nonionized form of both lidocaine and mepivacaine. Such increase in the lipophilic fraction of the local anaesthetic in turn increases the fraction of drug that can enter the neurons, which contributes to the faster onset of anaesthesia that is described with alkaline solutions. More important, the eased passage of local anaesthetics across neuron membranes reduces pain transmission, thereby lowering pain perception associated with tissue infiltration.

A reduction in pain at the site of injection, obtained through the addition of bicarbonate to lidocaine or mepivacaine, has been demonstrated in several clinical trials where local anaesthetics were given as cutaneous infiltration, including the treatment of traumatic wound lacerations [9], anaesthesia for digital nerve block [10], skin infiltration and intravenous catheterization prior to surgery [11], surgery for correction of prominent ears [12], repair surgery for combined upper eyelid blepharoplasty and levator advancement ptosis [13], carpal tunnel surgery [14, 15], and ambulatory phlebectomy procedures, the latter study testing specifically the association between mepivacaine and bicarbonate [16]. Overall, these studies were randomized controlled trials comparing a control group receiving the local anaesthetic given alone versus the group receiving the anaesthetic in a bicarbonate vehicle, and pain scores were obtained through visual analogic scales.

## 5. Conclusions

Both pharmaceutical and clinical evidence supports the use of sodium bicarbonate as a vehicle to dilute Akis and local anaesthetic (lidocaine and mepivacaine primarily) mixtures for subcutaneous injection.

## Figures and Tables

**Table 1:** A final injection volume of 5 mL was taken being fixed for all combinations tested.

Akis (final dilution in brackets)	Vehicle	Anaesthetic (final dilution in brackets)
25 mg/mL, 1 mL (5 mg/mL)	Normal saline 3 mL	Lidocaine 1% 1 mL (0.2%)
	Normal saline 2 mL	Lidocaine 1% 2 mL (0.4%)
	Water 3 mL	Lidocaine 1% 1 mL (0.2%)
	Water 2 mL	Lidocaine 1% 2 mL (0.4%)
	Bicarbonate 3 mL	Lidocaine 1% 1 mL (0.2%)
	Bicarbonate 2 mL	Lidocaine 1% 2 mL (0.4%)
	Normal saline 3 mL	Lidocaine 2% 1 mL (0.4%)
	Normal saline 2 mL	Lidocaine 2% 2 mL (0.8%)
	Water 3 mL	Lidocaine 2% 1 mL (0.4%)
	Water 2 mL	Lidocaine 2% 2 mL (0.8%)
	Bicarbonate 3 mL	Lidocaine 2% 1 mL (0.4%)
	Bicarbonate 2 mL	Lidocaine 2% 2 mL (0.8%)

50 mg/mL, 1 mL (10 mg/mL)	Normal saline 3 mL	Lidocaine 1% 1 mL (0.2%)
	Normal saline 2 mL	Lidocaine 1% 2 mL (0.4%)
	Water 3 mL	Lidocaine 1% 1 mL (0.2%)
	Water 2 mL	Lidocaine 1% 2 mL (0.4%)
	Bicarbonate 3 mL	Lidocaine 1% 1 mL (0.2%)
	Bicarbonate 2 mL	Lidocaine 1% 2 mL (0.4%)
	Normal saline 3 mL	Lidocaine 2% 1 mL (0.4%)
	Normal saline 2 mL	Lidocaine 2% 2 mL (0.8%)
	Water 3 mL	Lidocaine 2% 1 mL (0.4%)
	Water 2 mL	Lidocaine 2% 2 mL (0.8%)
	Bicarbonate 3 mL	Lidocaine 2% 1 mL (0.4%)
	Bicarbonate 2 mL	Lidocaine 2% 2 mL (0.8%)

25 mg/mL, 1 mL (5 mg/mL)	Normal saline 3 mL	Mepivacaine 1% 1 mL (0.2%)^*∗*^
	Normal saline 2 mL	Mepivacaine 1% 2 mL (0.4%)^*∗*^
	Water 3 mL	Mepivacaine 1% 1 mL (0.2%)^*∗*^
	Water 2 mL	Mepivacaine 1% 2 mL (0.4%)^*∗*^
	Bicarbonate 3 mL	Mepivacaine 1% 1 mL (0.2%)^*∗*^
	Bicarbonate 2 mL	Mepivacaine 1% 2 mL (0.4%)^*∗*^
	Normal saline 3 mL	Mepivacaine 2% 1 mL (0.4%)
	Normal saline 2 mL	Mepivacaine 2% 2 mL (0.8%)
	Water 3 mL	Mepivacaine 2% 1 mL (0.4%)
	Water 2 mL	Mepivacaine 2% 2 mL (0.8%)
	Bicarbonate 3 mL	Mepivacaine 2% 1 mL (0.4%)
	Bicarbonate 2 mL	Mepivacaine 2% 2 mL (0.8%)

50 mg/mL, 1 mL (10 mg/mL)	Normal saline 3 mL	Mepivacaine 1% 1 mL (0.2%)^*∗*^
	Normal saline 2 mL	Mepivacaine 1% 2 mL (0.4%)^*∗*^
	Water 3 mL	Mepivacaine 1% 1 mL (0.2%)^*∗*^
	Water 2 mL	Mepivacaine 1% 2 mL (0.4%)^*∗*^
	Bicarbonate 3 mL	Mepivacaine 1% 1 mL (0.2%)^*∗*^
	Bicarbonate 2 mL	Mepivacaine 1% 2 mL (0.4%)^*∗*^
	Normal saline 3 mL	Mepivacaine 2% 1 mL (0.4%)
	Normal saline 2 mL	Mepivacaine 2% 2 mL (0.8%)
	Water 3 mL	Mepivacaine 2% 1 mL (0.4%)
	Water 2 mL	Mepivacaine 2% 2 mL (0.8%)
	Bicarbonate 3 mL	Mepivacaine 2% 1 mL (0.4%)
	Bicarbonate 2 mL	Mepivacaine 2% 2 mL (0.8%)

25 mg/mL, 1 mL (5 mg/mL)	Normal saline 3 mL	Bupivacaine 0.25% 1 mL (0.05%)^*∗*^
	Normal saline 2 mL	Bupivacaine 0.25% 2 mL (0.1%)^*∗*^
	Water 3 mL	Bupivacaine 0.25% 1 mL (0.05%)^*∗*^
	Water 2 mL	Bupivacaine 0.25% 2 mL (0.1%)^*∗*^
	Bicarbonate 3 mL	Bupivacaine 0.25% 1 mL (0.05%)^*∗*^
	Bicarbonate 2 mL	Bupivacaine 0.25% 2 mL (0.1%)^*∗*^
	Normal saline 3 mL	Bupivacaine 0.5% 1 mL (0.1%)
	Normal saline 2 mL	Bupivacaine 0.5% 2 mL (0.2%)
	Water 3 mL	Bupivacaine 0.5% 1 mL (0.1%)
	Water 2 mL	Bupivacaine 0.5% 2 mL (0.2%)
	Bicarbonate 3 mL	Bupivacaine 0.5% 1 mL (0.1%)
	Bicarbonate 2 mL	Bupivacaine 0.5% 2 mL (0.2%)

50 mg/mL, 1 mL (10 mg/mL)	Normal saline 3 mL	Bupivacaine 0.25% 1 mL (0.05%)^*∗*^
	Normal saline 2 mL	Bupivacaine 0.25% 2 mL (0.1%)^*∗*^
	Water 3 mL	Bupivacaine 0.25% 1 mL (0.05%)^*∗*^
	Water 2 mL	Bupivacaine 0.25% 2 mL (0.1%)^*∗*^
	Bicarbonate 3 mL	Bupivacaine 0.25% 1 mL (0.05%)^*∗*^
	Bicarbonate 2 mL	Bupivacaine 0.25% 2 mL (0.1%)^*∗*^
	Normal saline 3 mL	Bupivacaine 0.5% 1 mL (0.1%)
	Normal saline 2 mL	Bupivacaine 0.5% 2 mL (0.2%)
	Water 3 mL	Bupivacaine 0.5% 1 mL (0.1%)
	Water 2 mL	Bupivacaine 0.5% 2 mL (0.2%)
	Bicarbonate 3 mL	Bupivacaine 0.5% 1 mL (0.1%)
	Bicarbonate 2 mL	Bupivacaine 0.5% 2 mL (0.2%)

25 mg/mL, 1 mL (5 mg/mL)	Normal saline 3 mL	Levobupivacaine 0.25% 1 mL (0.05%)
	Normal saline 2 mL	Levobupivacaine 0.25% 2 mL (0.1%)
	Water 3 mL	Levobupivacaine 0.25% 1 mL (0.05%)
	Water 2 mL	Levobupivacaine 0.25% 2 mL (0.1%)
	Bicarbonate 3 mL	Levobupivacaine 0.25% 1 mL (0.05%)
	Bicarbonate 2 mL	Levobupivacaine 0.25% 2 mL (0.1%)
	Normal saline 3 mL	Levobupivacaine 0.75% 1 mL (0.15%)
	Normal saline 2 mL	Levobupivacaine 0.75% 2 mL (0.3%)
	Water 3 mL	Levobupivacaine 0.75% 1 mL (0.15%)
	Water 2 mL	Levobupivacaine 0.75% 2 mL (0.3%)
	Bicarbonate 3 mL	Levobupivacaine 0.75% 1 mL (0.15%)
	Bicarbonate 2 mL	Levobupivacaine 0.75% 2 mL (0.3%)

50 mg/mL, 1 mL (10 mg/mL)	Normal saline 3 mL	Levobupivacaine 0.25% 1 mL (0.05%)
	Normal saline 2 mL	Levobupivacaine 0.25% 2 mL (0.1%)
	Water 3 mL	Levobupivacaine 0.25% 1 mL (0.05%)
	Water 2 mL	Levobupivacaine 0.25% 2 mL (0.1%)
	Bicarbonate 3 mL	Levobupivacaine 0.25% 1 mL (0.05%)
	Bicarbonate 2 mL	Levobupivacaine 0.25% 2 mL (0.1%)
	Normal saline 3 mL	Levobupivacaine 0.75% 1 mL (0.15%)
	Normal saline 2 mL	Levobupivacaine 0.75% 2 mL (0.3%)
	Water 3 mL	Levobupivacaine 0.75% 1 mL (0.15%)
	Water 2 mL	Levobupivacaine 0.75% 2 mL (0.3%)
	Bicarbonate 3 mL	Levobupivacaine 0.75% 1 mL (0.15%)
	Bicarbonate 2 mL	Levobupivacaine 0.75% 2 mL (0.3%)

25 mg/mL, 1 mL (5 mg/mL)	Normal saline 3 mL	Ropivacaine 0.2% 1 mL (0.04%)
	Normal saline 2 mL	Ropivacaine 0.2% 2 mL (0.08%)
	Water 3 mL	Ropivacaine 0.2% 1 mL (0.04%)
	Water 2 mL	Ropivacaine 0.2% 2 mL (0.08%)
	Bicarbonate 3 mL	Ropivacaine 0.2% 1 mL (0.04%)
	Bicarbonate 2 mL	Ropivacaine 0.2% 2 mL (0.08%)
	Normal saline 3 mL	Ropivacaine 1% 1 mL (0.2%)
	Normal saline 2 mL	Ropivacaine 1% 2 mL (0.4%)
	Water 3 mL	Ropivacaine 1% 1 mL (0.2%)
	Water 2 mL	Ropivacaine 1% 2 mL (0.4%)
	Bicarbonate 3 mL	Ropivacaine 1% 1 mL (0.2%)
	Bicarbonate 2 mL	Ropivacaine 1% 2 mL (0.4%)

50 mg/mL, 1 mL (10 mg/mL)	Normal saline 3 mL	Ropivacaine 0.2% 1 mL (0.04%)
	Normal saline 2 mL	Ropivacaine 0.2% 2 mL (0.08%)
	Water 3 mL	Ropivacaine 0.2% 1 mL (0.04%)
	Water 2 mL	Ropivacaine 0.2% 2 mL (0.08%)
	Bicarbonate 3 mL	Ropivacaine 0.2% 1 mL (0.04%)
	Bicarbonate 2 mL	Ropivacaine 0.2% 2 mL (0.08%)
	Normal saline 3 mL	Ropivacaine 1% 1 mL (0.2%)
	Normal saline 2 mL	Ropivacaine 1% 2 mL (0.4%)
	Water 3 mL	Ropivacaine 1% 1 mL (0.2%)
	Water 2 mL	Ropivacaine 1% 2 mL (0.4%)
	Bicarbonate 3 mL	Ropivacaine 1% 1 mL (0.2%)
	Bicarbonate 2 mL	Ropivacaine 1% 2 mL (0.4%)

^*∗*^To our knowledge, mepivacaine 1% and bupivacaine 0.25% formulation should be available on the market, although we were unable to obtain them. Therefore, stock solutions of mepivacaine 1% and bupivacaine 0.25% were prepared from mepivacaine hydrochloride 2% W/V [Mepicain 2% (Monico SPA)] and bupivacaine hydrochloride: 5 mg/mL (0.5% W/V) [Marcaina 5 mg/mL (Astra Zeneca)], respectively, by diluting 1 : 1 the commercially available solutions with the relevant vehicles.

**Table 2:** The table reports the Akis + anaesthetic combinations resulting in limpid or turbid solutions.

Akis (final dilution in brackets)	Vehicle	Anaesthetic (final dilution in brackets)	
25 mg/mL, 1 mL (5 mg/mL)	Bicarbonate 3 mL	Lidocaine 1% 1 mL (0.2%)	Limpid
	Bicarbonate 2 mL	Lidocaine 1% 2 mL (0.4%)	Limpid
	Bicarbonate 3 mL	Lidocaine 2% 1 mL (0.4%)	Limpid

50 mg/mL, 1 mL (10 mg/mL)	Bicarbonate 3 mL	Lidocaine 1% 1 mL (0.2%)	Limpid
	Bicarbonate 2 mL	Lidocaine 1% 2 mL (0.4%)	Limpid
	Bicarbonate 3 mL	Lidocaine 2% 1 mL (0.4%)	Limpid

25 mg/mL, 1 mL (5 mg/mL)	Bicarbonate 3 mL	Mepivacaine 1% 1 mL (0.2%)	Limpid
	Bicarbonate 2 mL	Mepivacaine 1% 2 mL (0.4%)	Limpid
	Bicarbonate 3 mL	Mepivacaine 2% 1 mL (0.4%)	Limpid

50 mg/mL, 1 mL (10 mg/mL)	Bicarbonate 3 mL	Mepivacaine 1% 1 mL (0.2%)	Limpid
	Bicarbonate 2 mL	Mepivacaine 1% 2 mL (0.4%)	Limpid
	Bicarbonate 3 mL	Mepivacaine 2% 1 mL (0.4%)	Limpid

25 mg/mL, 1 mL (5 mg/mL)	Bicarbonate 3 mL	Bupivacaine 0.25% 1 mL (0.05%)	Limpid
	Bicarbonate 3 mL	Bupivacaine 0.5% 1 mL (0.1%)	Turbid

50 mg/mL, 1 mL (10 mg/mL)	Bicarbonate 3 mL	Bupivacaine 0.25% 1 mL (0.05%)	Limpid
	Bicarbonate 2 mL	Bupivacaine 0.25% 2 mL (0.1%)	Turbid

25 mg/mL, 1 mL (5 mg/mL)	Normal saline 3 mL	Ropivacaine 0.2% 1 mL (0.04%)	Turbid
	Sterile water 3 mL	Ropivacaine 0.2% 1 mL (0.04%)	Turbid
	Bicarbonate 3 mL	Ropivacaine 0.2% 1 mL (0.4%)	Turbid

50 mg/mL, 1 mL (10 mg/mL)	Normal saline 3 mL	Ropivacaine 0.2% 1 mL (0.04%)	Turbid
	Sterile water 3 mL	Ropivacaine 0.2% 1 mL (0.04%)	Turbid
	Bicarbonate 3 mL	Ropivacaine 0.2% 1 mL (0.4%)	Turbid
